# Supplementary material for: Spatiotemporal Distribution Patterns of Osthole and Expression Correlation of the MOT1 Homologue in Cultivated Angelica biserrata
Source: Int J Mol Sci. 2025 Nov 5;26(21):10746. doi: 10.3390/ijms262110746 (PMC12609638; doi:10.3390/ijms262110746)
Supplement: Supplementary file 1 [file ijms-26-10746-s001.zip › supplement figure.pdf]

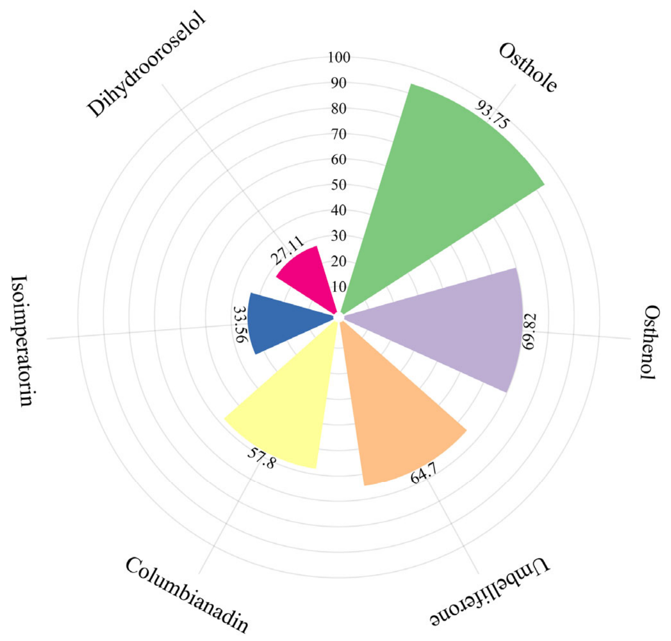

Figure S1. Coefficients of variation (CV) for six marker metabolites. Data calculated from three biological replicates (accessions P1, P2, P3) for each tissue type (root bark, root pith, petiole, and leaf).

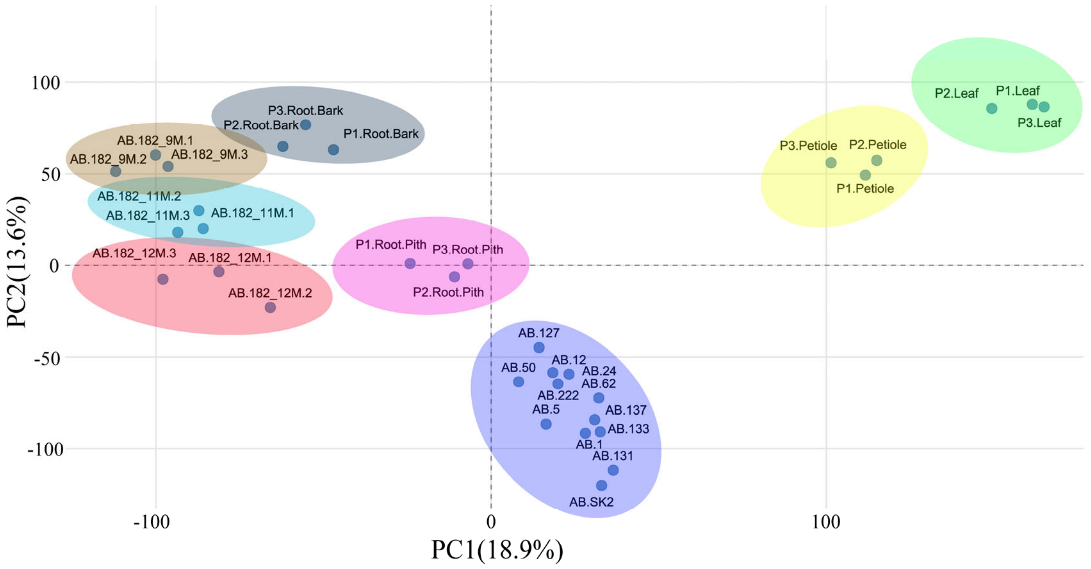

Figure S2. Principal Component Analysis (PCA) of transcriptome dataset. Principal components analysis of RNA-seq data from 33 biological samples. Variance explained by PC1 and PC2 is indicated on axes. Elliptical groupings represent statistically defined clusters (95% confidence interval) corresponding to sample types.

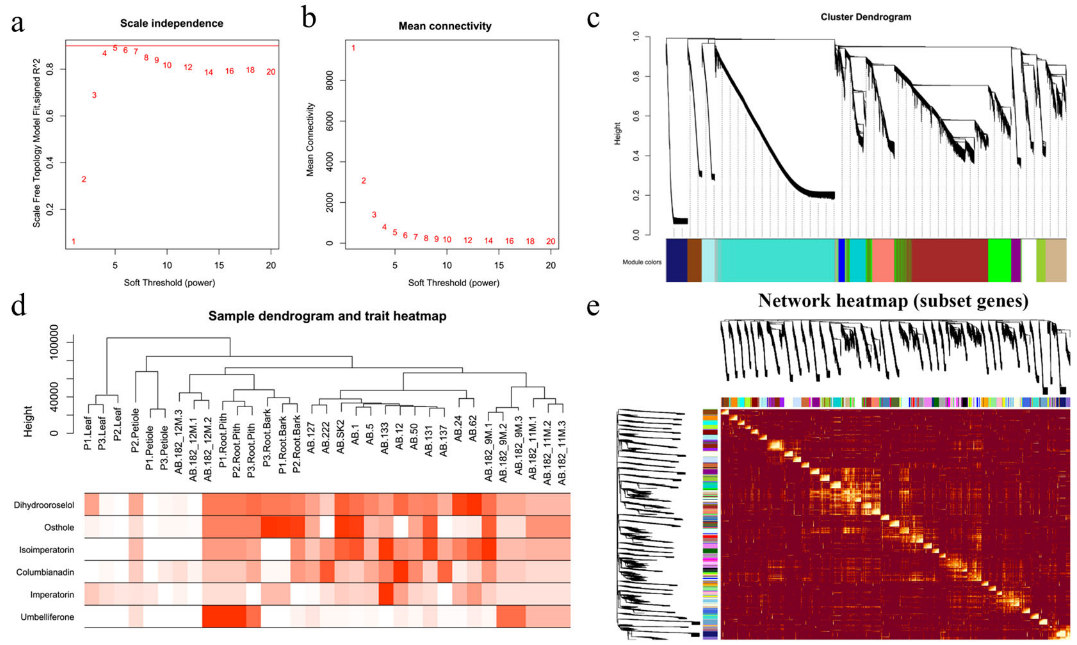

Figure S3. WGCNA workflow and network diagnostics. (a) Scale-free topology fit index analysis for soft-thresholding power selection. Red horizontal line indicates scale-free topology model fit (R^2=0.95). Optimal power value (power=4) denoted by dashed vertical line. (b) Mean connectivity analysis corresponding to soft-thresholding powers. Sharp decline cessation identifies network preservation threshold (power=4). (c) Hierarchical clustering dendrogram with module color assignment (DynamicTreeCut algorithm, minModuleSize=200). Resulting 44 co-expression modules are represented by distinct color bands. (d) Sample cluster dendrogram (top) with trait heatmap correlation matrix (bottom). Trait associations include six coumarin metabolites and spatial/temporal sample attributes. (e) Topological overlap matrix heatmap visualization of all detected modules. Diagonal pattern demonstrates high intramodular connectivity. Color key indicates topological overlap (TOM) values from 0 (yellow) to 1 (red).

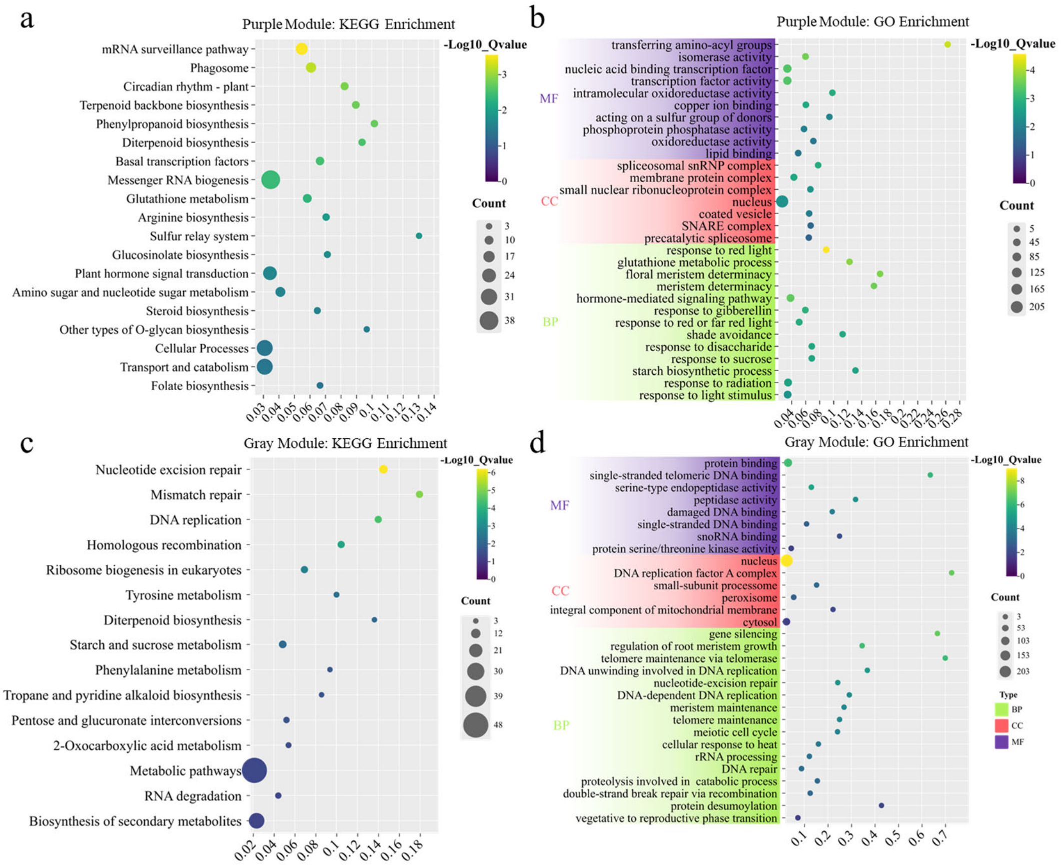

Figure S4. Functional enrichment analysis of the WGCNA purple module co-expressed genes. (a) KEGG pathway enrichment bubble plot of purple module genes showing significantly enriched pathways, where bubble size corresponds to gene count per pathway and color intensity indicates (adjusted q-value). (b) Gene Ontology (GO) enrichment bubble plot of purple module genes categorizing terms by hierarchical domain: Molecular Function (MF) shown in purple, Cellular Component (CC) in red, and Biological Process (BP) in green, with bubble size representing gene count per term. (c) KEGG pathway enrichment analysis of gray module genes (correlation with osthol: r = -0.0027) showing no significant enrichment in osthol biosynthesis-related pathways. (d) GO enrichment analysis of gray module genes displaying no significant enrichment in terms related to osthol biosynthesis.

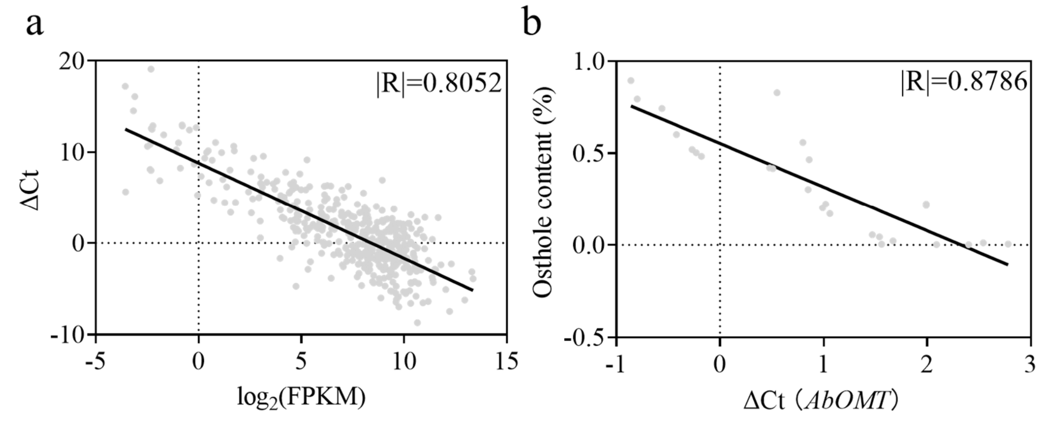

Figure S5. Expression pattern validation and product correlation analysis based on diverse Angelica biserrata root samples. (a) Scatter plot of $\log_2$(FPKM) derived from RNA-seq versus ΔCt values from qRT-PCR quantification of 31 selected genes across multiple samples (grey points, n=434) with fitted linear regression curve (black line; |R| = 0.8052). (b) Scatter plot of *AbOMT* gene expression measured by qRT-PCR (ΔCt values) versus corresponding Osthole accumulation intensity (% content) in different samples (grey points, n=26) with fitted linear regression curve (black line; |R| = 0.8786).
